# Lectures

**Published:** 1844-08-10

**Authors:** Benjamin Collins Brodie

**Affiliations:** Consulting-Surgeon of the Hospital


### CLINICAL LECTURES AND REPORTS.

### LECTURES

*Delivered in the Theatre of St. George's Hospital, in the session* 1843-44,

BY SIR BENJAMIN COLLINS BRODIE,

Consulting-Surgeon of the Hospital.

### EXTRACTION OF FOREIGN BODIES.

*Extraction of foreign bodies continued. Foreign substances in the large intestines. Removal of scybalæ. Bodies in the urinary organs. Effects of balls and shot in the human body. Of pins and needles—remarkable case. Foreign bodies in the trachea. Operation of tracheotomy.*

Gentlemen,—I mentioned, at the conclusion of my last lecture, that foreign bodies taken into the mouth not unfrequently stop in the rectum; but they may get into the rectum in other ways. Mr. Thomas was sent for to a gentleman under the following circumstances:—He had been very subject to costive bowels, and he used to make them act by introducing a piece of stick or cane eight or ten inches in length into the rectum, and there he left it, until, irritating the mucous membrane of the intestines, they acted, answering the purpose of an injection. He had been in the habit of doing this for some years, but one day the cane slipped out of his hand, and, to use his own expression, "it was sucked up into the gut." At first he was ashamed to send to Mr. Thomas, but after it had been there some days, such was the torture that he sent for him in great distress. Mr. Thomas introduced his finger into the rectum, but he could feel nothing. The sphincter muscle gradually relaxed, and he was then able to get in two fingers, and in a few minutes he passed in his whole hand. He then felt the piece of cane sticking obliquely at the upper part of the gut, and he abstracted it without any mischief. There is, in this case, a circumstance of great interest, and one that I believe was first observed by Mr. Thomas, namely, that the sphincter muscle gradually became relaxed under the pressure of the hand, so as to admit not only one finger, but two, and ultimately the whole hand. I have observed the same thing in several cases in which I have had occasion to make an examination, and the knowledge of this fact is very useful indeed on certain occasions which occur not only in hospital, but not unfrequently in private practice. I am very glad to have an opportunity of explaining to you the cases to which I allude, because I remember well that when I first met with them in private practice they puzzled me very much, and I shall be glad if you are saved that perplexity which I suffered myself. Persons of the affluent classes, for the most part, attend a great deal to the state of their bowels, and it is necessary that they should all do so. Those who live luxuriant and indolent lives are liable to have their bowels become very torpid, and you may be assured that there is no harm in their constantly attending to their bowels. I have known people belonging to the affluent classes who have been in the habit of taking medicine almost every day. I know one hearty old gentleman, eighty-six years of age, who can walk round the Regent's-park, who has taken an aloetic pill every night for threescore years. I knew another gentleman, who died at ninety-two, who took either an aloetic or a rhubarb pill for the same length of time, and I could give many other examples. But there are others who do not attend to their bowels; scybalæ form in the colon, they pass on to the rectum, but they are not easily discharged per anum. The softer fæces pass over the scybalæ, other scybalæ descend into the rectum, and the accumulation goes on until at last the rectum becomes completely filled up with a great mass of hardened fæces, as large as the fist, and even larger, so that half a pound, or perhaps a pound weight, may be collected there. The patient now suffers exceedingly, and he—or perhaps I ought to say she, for it is more common in women than in men—has desire to go to the water-closet. She goes, great pain is produced, but nothing comes away, the bowels being stopped up with these hardened fæces. The nature of the complaint may be ascertained by introducing the finger into the rectum; you there feel the hard mass of fæces. How is that to be got rid of? By injection? An injection will not act on this large mass. You must first dilate the sphincter muscle by introducing the fingers, and then with the handle of one or two pretty large spoons the whole mass may be extracted. A good nurse can accomplish it very well, if you tell her how. Let her take a couple of dessert-spoons and bring away a little and a little more, and when the rectum is nearly empty, warm water injected two or three times will remove the remainder. Until I was aware how much the sphincter muscle might be dilated I found it difficult to manage these cases. I used to try to accomplish it by introducing a narrow spoon into the rectum and bringing away a little at a time, but that was a very tedious process.

Foreign bodies may find their way into the urinary organs, and actually into the bladder. There is in the museum of the hospital a preparation of a calculus, which I purchased at the sale of the late Mr. Heaviside's museum. It is a section of a calculus formed upon a hazel-nut. It was extracted from the body of a woman by operation, and on cutting through the calculus the hazel-nut was found in the centre. There was no history of the case, but it is evident that the woman, playing some foolish trick with herself, had forced the hazel-nut through the urethra into the bladder. Mr. Thomas gives an account of a case in which he extracted a silver toothpick from the bladder. A woman had some difficulty in making water—probably an hysterical difficulty—she introduced the toothpick into the urethra, and it slipped back into the bladder. Sir A. Cooper cut a woman for what was supposed to be stone in the bladder, and when he removed it, it was found to be a piece of coal which had been thrust up the urethra.

No doubt these things are generally done from that peculiar perversion of mind which you find in very hysterical women; but it is sometimes done as a mere cheat, for the purpose of exciting compassion, and obtaining money from compassionate persons. A woman at Ryde, in the Isle of Wight, consulted Mr. Bloxam, a gentleman educated at this hospital, and who now resides there, for stone in the bladder. He introduced a pair of forceps and removed a stone of a very peculiar kind. By and by she had another, and he removed that. He thought they were very odd-looking stones, and as I happened to be at the Isle of Wight he showed them me, and told me that the woman had then got a third. We examined them, and they were evidently pieces of common lime stone, that the woman had cut into such a shape that she could push them into the bladder. She found it a good trade, inasmuch as she obtained money from the compassionate ladies of Ryde on account of her sufferings. I brought the stones up to town, and Dr. Prout examined them. Here the stones were really passed into the bladder; but I may take this opportunity of stating, by way of guarding you against what occurs in private practice, and, indeed, in hospital practice, that very frequently people pretend to pass calculi from the bladder which were never there at all. It is often very difficult to understand what motive there can be in women for trying to deceive in this respect. We can only attribute it to that perverted state of mind which I mentioned before, and which frequently amounts to insanity. Mr. Childer long ago—for he has now been dead twenty years— brought me a wafer-box full of what were said to be calculi passed from a young lady's bladder. On looking at them I said, "Calculi! they are bits of brick-bat and flint, and nothing else." He replied. "It is true, but there is a singular history belonging to them." He then told me this story:—A young lady, the daughter of a gentleman of fortune, all at once began to bleed, and, as she said, passed these calculi from the bladder. Her father and mother went to stay at the house of a country gentleman, and there she was taken very ill indeed at the water-closet, discharged a great quantity of blood, and produced an imense quantity of calculi, which she said came from the bladder; but they were examined very carefully, and found to be just what I have stated. I might mention many circumstances of the same kind. Among poorer people it is sometimes done for the sake of exciting compassion. A woman produced her little boy who was said to pass stones from the bladder. They were sent to me to examine, and I found that they were nothing but pebbles and flint. It was evidently a trick to get money from compassionate ladies, and in which she was successful.

But foreign substances find their way into the urinary organs of the male as well as the female.

A man came here with symptoms of stone, but on passing the sound the stone was felt anterior to the bladder. Sir Everard Home cut him for the stone, and brought out one that was narrow, but two or three inches in length. On making a section it was found to be formed on a flower-stalk, The history of his case was this:—The man was a gardener in the country; he had a stricture of the urethra, there was difficulty in making water, and occasionally he used to pass a flower-stalk as a bougie and relieve himself. One day the flower-stalk broke, it remained in, and formed the nucleus of a stone, half in the bladder and half in the urethra. I operated on a young man for stone in the bladder, and on cutting through the stone there was a large piece of common wax in the centre. The preparation, I believe, is in the museum. This was a very foolish young man, as you may suppose, who happened unluckily for himself, to have a wide urethra, and in some fit of folly he rolled up a piece of wax, introduced it into the urethra, and it gradually found its way back to the bladder. I saw him at the time, and, as I supposed that the wax had gone into the bladder, I recommended him to keep quiet, and let the case be thoroughly investigated. But he was engaged to go to India; he did not suffer inconvenience, as if from the wax in the bladder, though we had a right to conclude it was there, and, contrary to my advice to keep himself quiet, he sailed for India. He came back two years afterwards with a stone in his bladder, A more extraordinary case occurred in the practice of Mr. Keate: I saw the patient with him, and assisted in the operation. A gentleman had symptoms of stone in the bladder, and on cutting into that organ he found that there was no stone, but a great piece of common sealing-wax, of which he drew out several inches in length. This monstrous blockhead—for so I must call him—being tipsy, thought he would pass a bougie for himself. He imagined that wax was wanted for a bougie; he therefore procured the sealing-wax, softened it by the fire, rolled it up in his hands into the shape of a bougie, introduced twelve or thirteen inches through the urethra into the bladder, and there it lay ceiled up.

Foreign bodies may get into other parts of the human frame. A musket ball, for instance, may lodge in it for many years, doing no harm. A gentleman of my acquaintance was wounded the day before the battle of Waterloo. There was the hole at which it entered, but none at which it appeared to have escaped, so that it was no doubt lodged within. After a time the wound healed, and he got well. He was a young man, he frequented balls, danced like other people, and felt no inconvenience from the ball. He died several years afterwards of disease of the brain. But at other times musket-balls lodging in the human body may do great mischief. In the museum is a section of a diseased elbow which was amputated here. On sawing through it longitudinally a musket-ball was found in the centre of the bone, which had produced the disease of the joint. But musket-balls, even when lodged in soft parts, do harm in another way. A gentleman was shot in the eyes with small shot, and it produced the most dreadful case of neuralgia that I ever met with. I presume that the shot pressed on the optic nerve. A gentleman had a musket-ball lodge in the leg. It could not be felt, and as it gave him no inconvenience it might be doubted whether it was there or not. By and by it shifted its place, and became more superficial so as to be felt under the skin. Spasm now occurred in the leg, followed by fits resembling those of epilepsy, and to these he was subjected while the ball remained in this position. After a time the ball again shifted its place, went back, so that it could not be perceived externally, and then there was an end of the fits and of the other nervous symptoms. I presume that when it first shifted its place it pressed upon some nerve, and produced the spasm and these fits. Unfortunately, when it was in this situation, and might have been extracted, that course was not pursued; and when it again receded it would have been in vain to attempt it. It was of no use to look for a ball that you could not feel externally.

It is not uncommon for pins and needles to be found lying in the cellular membrane. Sir Charles Bell describes the case of a woman who had an abcess in her chest, from which was extracted a pin some two or three inches in length. It was supposed that the pin had been swallowed, and had made its way out through the œsophagus into the cellular membrane. In other cases it has been supposed that a pin or a needle which has been swallowed, has worked its way through the œsophagus into the chest or neck. But cases sometimes occur in which needles are taken out of human bodies in large numbers. The following case occurred in my practice:—A lady of hysterical habit was unfortunately married to a gentleman who became insane. Once or twice during a paroxysm he very nearly murdered her. What with anxiety about him, and apprehension about herself, her nervous system, which was bad enough to commence with, became much shaken. He died, but she remained in a frightful state,—very weak in health, with constant nervous pain on one side, and subject to what are called fainting fits; in fact, a sort of hysterical catalepsy,—that kind of fit which is produced by animal magnetism working on the imagination of hysterical women, in which the patient appears to be unconscious, but is not so in reality. One day she had with her a paper of needles, containing about fifty, fresh from the place where they were bought. She was by herself, she rang the bell in haste for the servant, and said that she had had one of her fits, and that the needles had run into her leg. This seemed a very odd story. Only eight needles out of the fifty were found left in the paper. It was thought that they had got into the footstool. That was unpicked, but nothing was found in it, except a few broken pins. They looked at her leg, and seeing something they did not understand, they sent for a surgeon in the neighbourhood, who found one or two needles pricking under the skin; he opened the skin with a lancet and took them out. In the course of two or three days other needles were discovered, he tried to take them out, but they slipped away, and I was sent for. With some trouble I removed them, and on a subsequent occasion I took out more; altogether we removed about twenty-eight needles from her leg—they were in one leg only. The leg became swollen and œdematous; and, having been in weak health before, she now became still weaker, and sank and died apparently from the mere want of nervous energy. On examining the leg I found several needles still left in it; they were not all taken out, but it would appear that there were just enough to account for those missing.

There are two points in this case to be considered; first, the taking out of the needles which, as a practical question, is of some importance; and, secondly, how the needles got there.

It may appear a very simple thing to extract needles that are stuck in a woman's leg, but it is not so simple in practice; for every motion of the limb makes the needles shift their situation; and if, in trying to remove them, you make any pressure upon them before you seize them with the forceps, they slip away. No attempt should be made to take needles out of the human body until they are close to the surface, and when you can with a light hand feel one end of them under the skin. You may then venture to puncture the skin with a lancet, and take care to pass, if possible, by the side of the needle, so as not to make pressure upon it. When you see the black point of the needle take hold of it with forceps and extract it. With a light hand you may take out a needle; but if a surgeon be rough, the needle slips away, and extraction is impossible.

But how was it that the needles in the case in question entered the leg. There is only one way of explaining it, namely, that she run them in herself. It is ridiculous to suppose that a paper of needles could run in by themselves. In this state of hysterical catalepsy the patients are not insensible. You know how the girls who are magnetised deceive and cheat. They pretend to read with the back of their head, and prophecy all sorts of stuff, and it is just the same here. This woman was humbugging herself in one way as they do in another. I have no doubt that she run the needles into her leg herself. I can conceive that one needle or two may run in by accident; you may sit down on some needles, and one or two may enter without your being aware of it at the time, but that a whole paper of needles could thus run in I do not believe. When a boy, I read in the "Annual Register" an account of an extraordinary case of a young woman who had swallowed a quantity of needles. The circumstance was forgotten, but years afterwards the needles made their appearance, and they were extracted, some from the arm, some from the breast, and some from other parts. This story was gravely recorded as one of needles having been swallowed, and then finding their way out of the stomach into different parts of the body many years afterwards. If a quantity of needles passed into the stomach I should think that they were more likely to do mischief to that organ itself, or to the intestines and peritoneum, than to run separately, and find their way out of the arms and legs. But I cannot understand how a woman could swallow twenty needles. Could you swallow twenty or thirty fish-bones? Certainly not. We know that hysterical women cheat in all manner of ways, and I have no doubt that these women run the needles in themselves. I do not, however, advise you, when called in, to expose such persons; for that is neither a kind nor a right thing to do. I have before said that some of the very best disposed young women will, when under the influence of hysterical disease, play tricks of this description. One young lady who, I believe, when in helth, was as good and honest as she could be, puzzled several medical men for a long time by mixing ink with her urine; and there are a number of stories of the same sort.

Foreign bodies may find their way into the trachea, and I shall conclude this lecture with a few observations on that subject.

A foreign body generally finds its way into the trachea in the following manner:—The patient has something in his mouth, he tries to speak just in the act of swallowing it, and in the effort to speak the epiglottis is raised just at the time when it ought to be shut down, and the morsel gets into the glottis. If it be large enough to stop the glottis it produces suffocation. It may, however, occasion coughing, and the cough generally brings it up, but at other times, instead of being coughed up, it slips down; that is, although the patient coughs, yet it slips down; and in other instances it slips down before he coughs,—and then you have a foreign body in the trachea.

The foreign bodies that thus enter the trachea may be very numerous and very various; for example, cherry-stones, almonds, pieces of meat, pieces of bone, gold and silver coin; and the effect they produce differs according to a variety of circumstances, according to their shape, and their particular position. The foreign body may be so large that it descends to the bifurcation of the trachea, and will not go down farther. It may be so large that it nearly fills up the diameter of the trachea, but that is not often the case, for a body that is small enough to go through the glottis will seldom be of sufficient size to fill up the trachea. Besides that, if it be broad it seldom lays directly flat across, but obliquely, and then there is a space on each side. Again, it may be very light, so that it rises up at every attempt to cough; or it may be very ponderous, so that it remains always at the most depending part. It may be small enough to pass down into one of the subdivisions of the trachea, and if it do it generally passes into the right bronchus, because that is the wider, and lies more nearly in a line with the trachea than the left. Supposing the body to be light, such as a cherry-stone or an almond, and smooth, and being smooth, moveable, it may lie at the bottom of the trachea, and the patient experience no inconvenience from it until a fit of coughing is excited. Several instances have occurred in which a small foreign body has been coughed up again through the glottis; and in other cases, being raised by the act of coughing, it has stuck in the glottis, strangled the patient, and produced instant death. But supposing that, without being very ponderous, it is of a large irregular shape, with sharp edges, and is lying across the trachea, with the corners stuck in one part of that passage, or of the bronchus; then it does not occasion at first much difficulty of breathing; for very probably there is sufficient space for the air to pass by its sides. But, being in the trachea, it brings on inflammation in the mucous membrane, attended with a great secretion of mucus; and this viscid mucus stops up that part of the opening of the trachea which is not blocked up by the foreign body, so that the tube becomes completely obstructed, and the patient dies of suffocation. This is another way in which it may prove fatal.

But supposing the foreign body to be composed of metal; that being a heavy substance, it will keep at the bottom of the trachea if it be of large size, or if small it will descend into the bronchus. Generally speaking it passes into the latter situation; for a metal body that is small enough to go through the aperture of the glottis will usually be small enough to be carried by its own weight to the bottom of the bronchus.

Another question arises here. What will happen when a ponderous body—a coin or other metallic substance—lodges in the bronchus? It will not cause great difficulty of breathing; for generally it is small enough not completely to obstruct that bronchus, or, at any rate, the patient can breathe with the other. It may occasion coughing, and if the patient invert himself so as to bring the head downward and elevate the chest, it will run down to the glottis and threaten suffocation. But the patient is in no danger of suffocation if he do not put himself into a position which not being a natural one, it is not likely that he will do. It may, therefore, remain lodged there for a long time; but what will happen at last? It will give rise to disease of the lungs. A man swallowed a Louis-d'or, which got into the trachea; he died three or four years afterwards, and on examination there was found disease in the lung, the Louis-d'or being in an abscess. This case is recorded in the "Memoirs of the French Academy of Surgery." A boy got a sixpence into the trachea; he was sent to Guy's Hospital, where it was found that his lungs were diseased, and therefore Mr. Key refused to make any attempt to remove the sixpence. A boy swallowed a tin tack; when I saw him he was expectorating pus, so that it had formed an abscess, and it was doubtful whether the tin tack were there or not. By and by he coughed up the tin tack, and the discharge of pus ceased, and the patient recovered.

Foreign bodies are very frequently coughed up, and it has been proposed to endeavour to expel them by making the patient cough. No doubt you can make the patient cough, and there is a chance of the foreign body being thus removed; certainly, if it is to be got rid of by natural efforts it must be by coughing. Some have proposed to bring up the foreign body by giving the patient an emetic, but I apprehend that it would be useless. In the act of vomiting there is a deep inspiration, not a forcible expiration; the diaphragm descends in order to press on the stomach, and, combining with the action of the abdominal muscles, it expels the contents of that organ. After vomiting is over there is no convulsive coughing, so that the diaphragm gradually returns to its own place. The idea of exhibiting an emetic is founded altogether on a wrong physiological notion; and I think it is dangerous to trust to the act of coughing for bringing up the foreign body. It is very true that it may be coughed up through the glottis; but it may stick in the glottis, and then the patient would die.

Now, what is the best rule to follow? If you are satisfied that the foreign body is in the trachea, I believe that the proper course to pursue is not to trust to nature; she may manage it, but you are not certain of it, and in a great number of cases where it is left to nature the patient dies. Make an opening in the trachea, and I believe it is best to make it low down. You may proceed here as leisurely as you please, for the patient is not in danger of instant suffocation. Take up every vessel as you proceed, and separate the parts as much as you can with a director, instead of cutting them, so as to avoid hæmorrhage. If you open the trachea when bleeding is going on, every time the patient inspires, blood is drawn into the trachea, and the patient may be suffocated by the surgeon opening the trachea too hastily, and allowing it to become filled with blood. I know a case in which a surgeon performed tracheotomy, and the patient died almost directly. On examining the body, as I am informed, the trachea and bronchi were full of blood. Make the opening, then, as leisurely as you please; separate the parts by a blunt instrument rather than a knife; divide three or four rings of the trachea longitudinally, but there is no occasion to remove any portion of the trachea. What will now occur? When you have divided the trachea, if it be a light and moveable body, such as a cherry or tamarind-stone, as soon as you have made the opening, if you hold back the edges, cough comes on, the foreign body is thrown up, and escapes by the artificial opening; or even if it do not escape there the danger of suffocation, in consequence of its sticking in the glottis, is prevented. But if the foreign body be a bone or any rough substance that is stuck in the trachea, and not moveable, then you may introduce the forceps and remove it; and I can conceive of cases in which it is right to take a foreign body from the bronchus. I advise you, however, never to attempt the latter if you can effect the object in any other way. The introduction of forceps into the bronchus will occasion violent coughing, great irritation, and it is a frightful thing to introduce these instruments into the bronchus when the patient is agitated by a convulsive cough. Only conceive of the important organs in the neighbourhood. The lungs are below, and you may injure them, or you may take hold of one of the subdivisions of the bronchi instead of the foreign body. There is the pulmonic plexus of nerves behind; you are close to the phrenic nerves; you are not far from the great vessels of the heart; and the heart itself is close by. Think of the mischief you may do by poking among these important organs with forceps when the patient is agitated by a convulsive cough. Still, if there be a piece of bone stuck across the bronchus, it may be the only way of taking it out. But it does not often happen that any ragged body will go into the bronchus; if, however, it do, you may have to introduce the forceps five or six inches from the part where you make the wound externally.

But suppose a case in which there is a loose and ponderous body in the bronchus. In the case of Mr. Brunel, which occurred last year, there was a half-sovereign in the right bronchus. This gentleman, in playing with a child, flung a half-sovereign into his mouth, and it slipped down the windpipe. In the first instance it produced sickness, and as he drew his breath, previously to vomiting, it descended into the bronchus and occasioned coughing every now and then. When his head was placed down it could be felt rolling along the trachea. We attempted to remove it by placing him on a moveable platform, so that his feet were up and his head down nearly at right angles. The half-sovereign descended and stuck in the glottis so as nearly to choke him. We, therefore, determined not to repeat this experiment till we had got an opening in the trachea which would act as a safety-valve. We made an opening some few days afterwards below the thyroid gland, but the half-sovereign was not coughed up as a cherry-stone would have been, because it was too heavy. We made some attempts to use the forceps, but found it so dangerous that we desisted. When he had recovered from the effects of this operation,—in the mean time passing a probe every now and then,—we again placed him on a moveable platform, his back was struck with the hand, and the half sovereign escaped from the bronchus. He could feel it rolling along the trachea till it came to the glottis, and now, instead of sticking there, it passed through, just as you could roll it through the dead body, and came out of the mouth. There was no spasm of the glottis, and the absence of it was to be attributed to the opening in the trachea; for blood came out with the half-sovereign which had evidently passed in from the external wound, and where blood went in you may be sure that the air went in also. I apprehend the rule to be this:—In all cases where a foreign body has got into the trachea you must not trust to nature, but make an opening into the trachea; and then it is very likely that if the body be light it will be forced through the opening; or if, by its own weight, it can be made to assume a certain position, it will pass out through the glottis; or, if it be a rough, irregular substance, and sticks in the trachea, you may then, through the artificial opening, seize it with the forceps and extract it. But I advise you to be very careful how you use the forceps, except where the foreign body is actually in the trachea; cases may occur in which you must use them in the bronchus, but it must be done with the greatest possible caution.—*Lond. Lancet.*

